# Estimation of Minimum Uncut Chip Thickness during Precision and Micro-Machining Processes of Various Materials—A Critical Review

**DOI:** 10.3390/ma15010059

**Published:** 2021-12-22

**Authors:** Szymon Wojciechowski

**Affiliations:** Faculty of Mechanical Engineering, Institute of Mechanical Technology, Poznan University of Technology, Piotrowo 3, 60-965 Poznan, Poland; szymon.wojciechowski@put.poznan.pl

**Keywords:** minimum uncut chip thickness, precision machining, ultra-precision machining, micro-machining

## Abstract

Evaluation of the phenomena characterizing the chip decohesion process during cutting is still a current problem in relation to precision, ultra-precision, and micro-machining processes of construction materials. The reliable estimation of minimum uncut chip thickness is an especially challenging task since it directly affects the machining process dynamics and formation of a surface topography. Therefore, in this work a critical review of the recent studies concerning the determination of minimum uncut chip thickness during precision, ultra-precision, and micro-cutting is presented. The first part of paper covers a characterization of the precision, ultra-precision, and micro-cutting processes. In the second part, the analytical, experimental, and numerical methods for minimum uncut chip thickness estimation are presented in detail. Finally, a summary of the research results for minimum uncut chip thickness estimation is presented, together with conclusions and a determination of further research directions.

## 1. Introduction

In 1983, Taniguchi [1] divided cutting technology into conventional, precision, and ultra-precision machining, taking into account the dimensional accuracy of the workpiece. Nevertheless, with the progress of time, the achievable dimensional deviations of the machined parts in the scope of the above-mentioned techniques have shifted to much lower values. According to the research of Dornfeld et al. [2], the reason for this trend is the growing demand for reduced weight and dimensions of products, improved surface quality, and dimensional accuracy, while reducing production costs.

Currently, according to the research [3,4,5,6], the use of conventional machining enables dimensional accuracy of the product *ε* > 0.8 µm to be obtained, and 0.008 µm < *ε* ≤ 0.8 µm in the case of precision machining. In the case of ultra-precision machining, the dimensional accuracy of the workpiece is in the range of 0.0008 µm < *ε* ≤ 0.008 µm. The division into conventional, precision, and ultra-precision machining can be made taking into account the ranges of the uncut chip thickness and considering the phenomena accompanying material decohesion.

According to the work of Dornfeld and Lee [7], in conventional machining, the uncut chip thickness is *h* ≥ 0.1 mm, in precision 1 µm < *h* < 0.1 mm, and in ultra-precision *h* ≤ 1 µm. In the case of conventional cutting, the chip formation process occurs as a result of material decohesion only in the shear zones. In the case of precision and ultra-precision machining, the chip formation phenomenon is also determined by the presence of a rounded radius of the cutting edge and by certain workpiece material characteristics overlooked by conventional machining. These factors primarily include micropores and fractures of the material, inclusions, grain boundaries, and interactions in dislocations (understood as physical interactions between different types of dislocations occurring in a plastically deformed material [8]), as well as the multi-phase structure of the workpiece.

Ultra-precision machining (UPM) includes machining techniques such as ultra-precision diamond turning (UPDT), ultra-precision raster milling (UPRM), ultra-precision grinding (UPG), and ultra-precision polishing (UPP) [9]. The main field of application of UPM techniques is the production of high-precision optical parts (e.g., digital camera lenses, CCD lenses, VCD, DVD). Due to the extremely high requirements for shape accuracy (in the range of 0.2 µm) and surface roughness (*Ra* < 10 nm) of the machined elements [10,11], the effective use of UPM techniques necessitates the use of diamond tools and ultra-precise machine tools. According to the Erdemir and Donnet [12], monocrystalline diamond (MCD) is the recommended tool material for machining with the use of UPM techniques due to the submicrometric contour of the tool and the nanometric radius of the cutting edge. In the case of ultra-precise machine tools, high accuracy, dynamic stiffness, and thermal stability are obtained through the use of drive elements in the form of air bearings, piezoelectric and friction drives, and high-resolution measuring elements (laser interferometers). Most often, the machine body is made of natural granite, polymer concrete, or cast iron in order to ensure high rigidity and vibration damping coefficient, and thus minimizing vibrations in the machining system [13].

**Figure 1:**
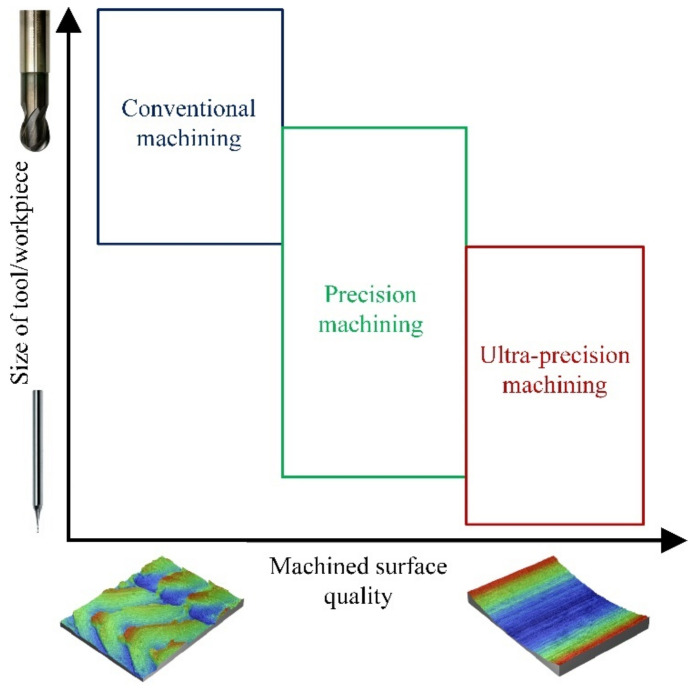
Area of application of conventional, precision, and ultra-precision machining in relation to the size of the workpiece and the cutting tool.

In recent years, the growing demand for the production of miniaturized parts with sizes ranging from several dozen micrometers to several millimeters has contributed to the increase in the popularity of so-called micro-machining. This technique mainly includes turning, drilling, and milling processes. It should be emphasized that micro-cutting processes can be implemented in the field of precision and ultra-precision machining (Figure 1).

Micro-milling is usually performed with the use of diamond tools [14] or solid carbide tools with diameters *D* ≤ 1 mm [15]. The smallest diameters of commercially produced cutters, with a non-zero helix angles, are 50 µm [16,17]. In the case of cutters with diameters less than 50 µm, a zero helix angle is used. This is justified by the production limitations, and the increased stiffness of the tool with the cutting edge with helix angle *λ_s_* = 0. Currently, the main area for micro-milling application is the production of components made of titanium alloys and stainless steels for the biomedical industry, such as bone and joint implants and parts for the neurovascular system [18]. This process is also used in the production of microelectrodes as well as microforms and microarrays from hardened alloy steels and elements of bio–micro–electro–mechanical systems (bio-MEMS) [19]. In addition, the use of single-edge diamond tools allows the formation of pyramidal or prismatic structures used in diffraction optics and light directing systems.

It follows from the above considerations that the scope of precision and ultra-precision machining can be defined by the dimensional accuracy and surface roughness of the workpiece and by the uncut chip thickness, as well as by the type and intensity of the phenomena occurring during the material decohesion. In the case of precision machining, the transformation of the area of cut into a chip takes place in the range of the uncut chip thicknesses comparable with the radius of the main cutting edge. Therefore, the chip formation phenomenon is determined not only by shear along the slip plane but also by elastic–plastic deformations of the workpiece and by certain material properties, such as, e.g., micropores and material fractures, inclusions, and grain boundaries. On the other hand, during ultra-precision machining, the decohesion phenomenon usually takes place in the range of the uncut chip thicknesses smaller than the cutting edge radius. The effect of this is the occurrence of intense ploughing of the material, as well as the influence of the interaction of dislocations in the structure and orientation of the crystals on the course of chip formation.

Based on the above deliberations one can conclude that the primary factor affecting the chip formation in precision, ultra-precision, and micro-machining processes is the minimum uncut chip thickness. Therefore, its reliable estimation is crucial in the characterization of phenomena appearing in machining processes.

**Figure 2:**
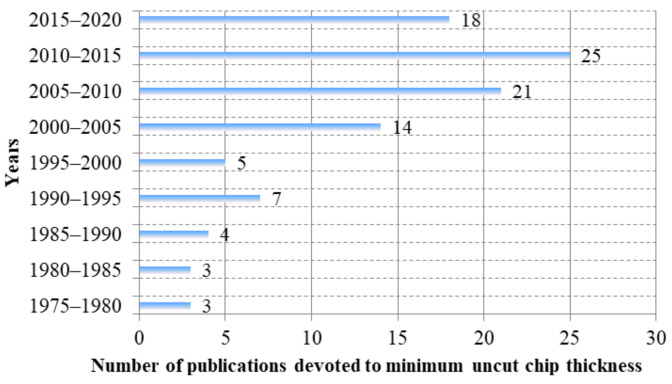
Number of publications devoted to minimum uncut chip thickness. Own elaboration.

Despite many experimental and theoretical studies on evaluation of minimum uncut chip thickness carried out in recent years (see Figure 2), a critical literature review has not yet been conducted. Therefore, the aim of this study was to carry out a comprehensive review of existing methods for the determination of minimum uncut chip thickness, particularly in precision, ultra-precision, and micro-machining processes. This work proposes a division of the minimum uncut chip thickness methods, as well as a discussion of the older (e.g., measurements of surface roughness, cutting forces, acoustic emission) and newer approaches (finite elements methods, molecular dynamics, smoothed particle hydrodynamics) applied for the evaluation of minimum uncut chip thickness.

## 2. General Division of Minimum Uncut Chip Thickness Estimation Methods

Converting the area of cut into a chip is a complex physical–chemical process involving the occurrence of elastic and plastic deformations, friction, heat generation and propagation, and tribological phenomena related to the progressive wear of the tool. Chip formation occurs as a result of material decohesion caused by a complex state of stress with a value exceeding the strength of the workpiece material. Depending on the mechanical properties of the material being cut, decohesion can occur as a brittle fracture (for brittle materials, e.g., glass, ceramics, sintered carbides) or plastic (for elastic–plastic materials, e.g., ferrous metals, non-ferrous metals, composites, plastics).

**Figure 3:**
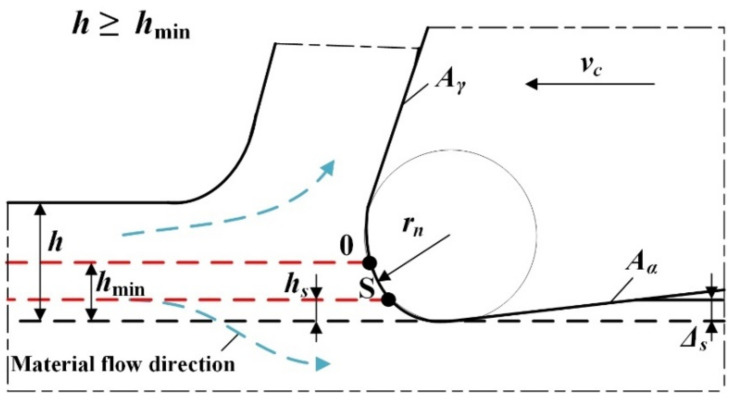
Model of orthogonal cutting with the uncut chip thickness contained in the range *h* ≥ *h*_min_ (*h* denotes the uncut chip thickness, *h*_min_ is the minimum uncut chip thickness, *h_s_* is the thickness of workpiece springback, Δ*s* is elastic deformation of workpiece, *v_c_* is cutting speed, *A_α_* is flank plane, *A_γ_* is rake plane, *r_n_* is cutting edge radius, 0 is the stagnant point, S is the point denoting the boundary of the workpiece springback). Own elaboration.

Due to the occurrence of elastic deformations of the workpiece and the non-zero radius of the main cutting edge *r_n_*, a certain volume of material flowing onto the cutting edge is not converted into a chip but is ploughed under the tool flank face *A_α_*. It should be emphasized that this phenomenon becomes important, especially in the field of precision machining, carried out with small thicknesses of the cut. In the case when the uncut chip thickness is smaller than the *h_s_* value (denoting the thickness of the workpiece springback), the workpiece is only subjected to elastic deformation with a value of Δ*s* and returns to the nominal position after the tool passes. Increasing the thickness of the uncut chip thickness to a value lower than *h*_min_ causes the formation of elastic–plastic and plastic deformations of the workpiece. The result is the formation of a plastic flash, which, however, is not transformed into a chip [20]. A further increase in the uncut chip thickness to the value within the range *h* ≥ *h*_min_ induces the cutting process and chip formation (Figure 3).

Based on the considerations presented above, it can be concluded that one of the most important factors characterizing the decohesion of the material is the *h*_min_ parameter. It is defined as the critical uncut chip thickness value that initiates the shearing and conversion of the workpiece into a chip.

Considering the geometric dependencies in the workpiece–cutting edge interface, the minimum uncut chip thickness could be defined by the following relation: *h*_min_ = *r_n_*. Nevertheless, in practice, the real value of minimum uncut chip thickness is usually lower than the cutting edge radius. According to Kawalec [21], the minimum uncut chip thickness depends on the cutting edge, cutting parameters, and properties of the workpiece and tool material. The above dependence can be described by the following equation:

(1) $$h_{\min}=k\cdot r_{n}$$

where: 

*h*_min_—the minimum uncut chip thickness;

*k*—normalized minimum uncut chip thickness;

*r_n_*—cutting edge radius.

The value of this coefficient depends on the mechanical properties of the workpiece and tool material, cutting parameters, and tribological phenomena at the contact between the workpiece and the tool.

**Figure 4:**
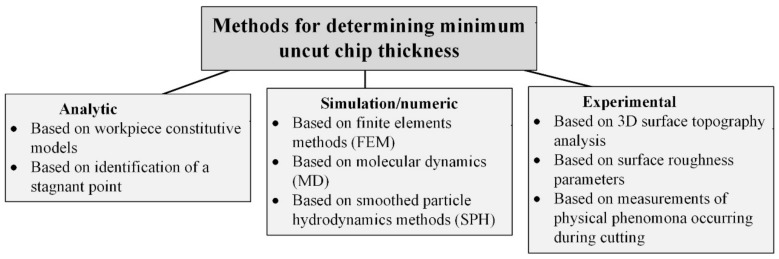
The division of minimum uncut chip thickness estimation methods. Own elaboration.

The intensively developing technology of precision, ultra-precision, and micro-machining has contributed to many studies including the development of measurement, computational, and numerical methods for determining *h*_min_. Therefore, the methods of estimating the minimum uncut chip thickness can be divided into three basic groups: analytical, simulation/numerical, and experimental (Figure 4).

## 3. Analytic Methods for Minimum Uncut Chip Thickness Determination

In analytic methods, the physical phenomena occurring during chip decohesion are taken into account. In order to describe these phenomena, a constitutive model of the workpiece or the identification methods of the material stagnant point are applied. 

In the model developed by Liu et al. [22], intended for micro-milling of steel and aluminum alloy, the transition between plastic deformation and micro-cutting in the scratch test is taken into account. According to the above model, the normalized minimum uncut chip thickness is formulated by the equation

(2) $$k=\frac{h_{\min}}{r_{n}}=0.5-\frac{\tau_{a}}{\tau_{sh}}$$

where: 

*τ_a_*—shear strength of adhesive joints;

*τ_sh_*—shear stress.

The values of the *τ_a_* and *σ* parameters contained in Equation (2) can be determined by using the so-called constitutive models. According to Jaspers and Dautzenberg [23], the Johnson–Cook [24] constitutive plasticity model can be successfully used to determine the value of the slip stress for aluminum and titanium alloys. However, for carbon steels its accuracy is limited. In the case of these materials, an alternative may be the Oxley constitutive model [25].

The Johnson–Cook model includes the shear stress *τ_sh_*, which is expressed as a function of temperature, deformation, and deformation speed of the workpiece. This equation can be formulated in the following form:

(3) $$\tau_{sh}=\left[A+B{\left(\varepsilon \right)}^{n_{\tau }}\right]\cdot \left[1+m_{\tau }\cdot \ln\left(\dot{\varepsilon }\right)\right]\cdot \left[1-{\left(\frac{T_{A}-T_{R}}{T_{T}-T_{R}}\right)}^{\nu_{\tau }}\right]$$

where: 

*A*, *B*—empiric constants;

*ε*—equivalent plastic deformation;

*n_τ_*—strengthening factor;

*m_τ_*—constant describing the sensitivity of the strain rate speed of plastic deformation;

*T_A_*—absolute temperature;

*T_R_*—reference temperature;

*T_T_*—melting temperature;

*υ_τ_*—thermal deformation coefficient.

In case of Oxley constitutive model, the value of shear stress is expressed in a form of the exponential strain function

(4) $$\tau_{sh}=\sigma_{1}\varepsilon^{n_{1}}$$

where: 

*σ*_1_—strength coefficient;

*n_1_*—exponent that takes into account the material hardening by plastic deformation.

The determination of the coefficients in Equations (3) and (4) is possible by applying the slip-line field theory developed by Liu et al. [26]. This approach applies to micro-machining processes because it takes into account heat sources coming from mechanical energy of shear along the slip plane and chip friction on the rake surface, as well as ploughing and friction phenomena in the area of the cutting edge radius. The analytic model of the minimum uncut chip thickness [26] has also been adapted by Ozel, Liu, and Dhanorker [27] to determine the minimum uncut chip thickness during end-milling of 4340 steel and Al 20204-T6 aluminum alloy, using a tool with a diameter of *D* = 0.635 mm. 

The knowledge of the shear stress during cutting enables estimation of the uncut chip thickness value corresponding to the thickness of the material springback *h_s_*. Jun et al. [28] adapted the quasi-static Johnson model [29] to determine the value of *h_s_* according to the equation

(5) $$h_{s}=0.5r_{n}{\left(\frac{2\tau_{sh}}{E}\right)}^{2}$$

where: 

*E*—Young modulus of the workpiece;

*h_s_*—thickness of material springback.

Another group of analytic models are approaches based on determining the so-called stagnant point 0 located on the rounded cutting edge. At this point, there is a change in the direction of flow of the workpiece relative to the tool. Therefore, the position of the 0 point is directly related to the minimum uncut chip thickness. It is worth indicating that the stagnant point is the boundary between the actual rake face and the actual tooth flank face. 

**Figure 5:**
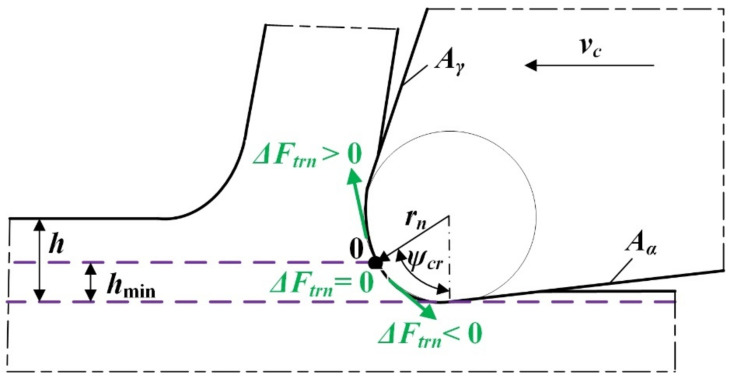
Determination of a stagnant point during orthogonal cutting (Δ*F_trn_* is tangential force at the stagnant point, *ψ_cr_* is stagnant angle). Own elaboration.

Storch and Zawada-Tomkiewicz [30] formulated an analytical *h_min_* model for orthogonal turning. This model is based on the analysis of the tangential forces *F_trn_* acting on the rounded cutting edge. The developed force models were expressed as a linear function of the uncut chip thickness. The authors assumed that the increase in tangential forces at the stagnant point is zero (Δ*F_trn_* = 0—Figure 5), which consequently made it possible to determine the stagnant angle *ψ_cr_* on the basis of the following equation:

(6) $$\psi_{\mathrm{cr}}=\mathrm{arcctg}\;\;\left(\frac{a_{f}}{a_{c}}\right)$$

where: 

*a_f_*, *a_c_*—directional coefficients in linear regression equations of feed force and cutting force;

*ψ_cr_*—stagnant angle.

Determining the expression describing the stagnant angle *ψ_cr_* allows for the formulation of the equation for the minimum uncut chip thickness

(7) $$h_{\min}=r_{n}\left(1-\cos\psi_{\mathrm{cr}}\right)=r_{n}\left[1-\cos\;\left(\mathrm{arcctg}\;\;\left(\frac{a_{f}}{a_{c}}\right)\right)\right]$$

Son et al. [31] formulated the *h_min_* model for orthogonal diamond micro-turning. They assumed that in the range of *h_min_*, the workpiece undergoes ideally elastic and plastic deformations. In order to define the expression for the minimum uncut chip thickness, they adapted the analytical Merchant force model and used the condition of the equilibrium of forces at the stagnant point. They obtained the expression for *h_min_* in the following form:

(8) $$h_{\min}=r_{n}\left(1-\cos\;\;\left(\frac{\pi }{4}-\frac{\Theta }{2}\right)\right)$$

where: 

*Θ*—The angle of friction of the chip against the rake face or the workpiece against the tool flank face.

The friction angle *Θ* in Equation (8) can be described by the equation

(9) Θ=arctg  μ

where: 

*µ*—coefficient of friction of the chip against the rake face or the workpiece against the tool flank face.

In a subsequent study, Son et al. [32] extended their analytical model to an ultrasonic-assisted precision machining process of aluminum, brass, and OFHC. The authors assumed that ultrasonic tool vibration in the cutting direction promotes an increase in the friction coefficient between a tool and a workpiece. Therefore, the authors formulated the following model of friction coefficient, required for the estimation of minimum uncut chip thickness during ultrasonic-assisted precision machining:

(10) $$\mu =\frac{H_{T}A_{T}}{H_{N}A_{N}}$$

where: 

*H_T_*, *H_N_*—tangential and normal hardness of a workpiece material; 

*A_T_*, *A_N_*—the tangential and normal cross sectional area of a tool–workpiece contact.

Based on the research, authors have indicated that independent of the type of workpiece material, the growth of tool vibration frequency has led to the growth of the friction coefficient and thus to the growth of minimum uncut chip thickness.

Malekian et al. [33] estimated the minimum uncut chip thickness in the precision orthogonal turning process. In order to identify the stagnant point, they assumed that this point corresponds to the occurrence of the minimum energy needed for the decohesion of the workpiece. The authors formulated the expression for the total cutting energy, taking into account the ploughing and shear forces. Then they found the derivative of the cutting energy due to the stagnant angle and equated it to zero. As a result of the transformations, they obtained the expression for the stagnation angle in the following form:

(11) $$\psi_{cr}\approx \Theta$$

Przestacki et al. [34] formulated the minimum uncut chip thickness model for the oblique cutting of WC/NiCr clad layers. In the oblique cutting process conducted with the rounded corner of insert, the instantaneous uncut chip thickness values are variable as a function of the width of the cut. It consequently influences the distribution of cutting force components and makes the estimation of minimum uncut chip thickness more complex. The authors assumed that the cutting force components and their increments (Δ*F_ci_*, Δ*F_fi_*, Δ*F_pi_*) are located in the center of gravity of the chip cross section. Subsequently, they formulated the following equations of force increments:

(12) $$\Delta F_{ci}=C_{c}\cdot \Delta h$$

(13) $$\Delta F_{fi}=C_{f}\cdot \Delta h$$

(14) $$\Delta F_{pi}=C_{p}\cdot \Delta h$$

where:

Δ*h* = *h_i_* − *h_i_*_−1_—the increment of uncut chip thickness; 

*C_c_*, *C_f_*, *C_p_*—empirical slope coefficients of the *F_c_*, *F_f_*, *F_p_* forces.

Ultimately, the minimum uncut chip thickness was calculated from the following equation:

(15) $$h_{\min}=r_{n}\left[1-\sin\left(\arctan\left(\frac{C_{f}\cdot \sin\varphi_{c}+C_{p}\cdot \cos\varphi_{c}}{C_{c}}\right)\right)\right]$$

where:

*φ_c_*—angle denoting the center of gravity of area of cut.

Wojciechowski [35] proposed a minimum uncut chip thickness model, dedicated to the micro-end milling process. Estimating the minimum uncut chip thickness for milling is a much more complex task than in the case of orthogonal free turning, during which the components of the total force and the values of the cross-sectional area of cut are relatively constant over time. The reason for this is the kinematics of milling, which determines the variability of the cross-sectional area of cut, and thus the value of the forces as a function of tool rotation angle. Therefore, the author proposed a method based on determining the mean values of the tangential and radial forces in the normal plane—*F_tn_av_*, *F_rn_av_*—and relating them to the mean values of the uncut chip thickness *h_z_av_*. It was assumed that during slot milling, average forces occur for the angle *φ* = π/2. Then the values of the force components *F_tn_av_*, *F_rn_av_* are calculated on the basis of the mean square forces values in the machine tool system according to equations

(16) $$\begin{array}{l} F_{tn\_\mathrm{av}}=F_{x\_RMS}\cdot \cos\lambda_{s}+F_{z\_RMS}\cdot \sin\lambda_{s}\\ F_{rn\_\mathrm{av}}=F_{y\_RMS} \end{array}$$

where:

*F_x_RMS_*, *F_y_ RMS_*, *F_z_ RMS_*—root-mean-square values of predicted/measured force components in machine tool coordinate system;

*F_tn_av_*, *F_rn_av_*—the mean values of the tangential and radial forces in the normal plane.

In the next stage, the regression equations of *F_tn_av_*, *F_rn_av_* forces, as a function of average uncut chip thickness, are being formulated from the equations

(17) $$\begin{array}{l} F_{tn}=a_{tn}\cdot h+F_{te}\\ F_{rn}=a_{rn}\cdot h+F_{re} \end{array}$$

where:

*a_tn_*, *a_rn_*—empirical slope coefficients of the force components, *F_te_*, *F_re_* are the edge forces;

*h*—uncut chip thickness.

In the last stage, the minimum uncut chip thickness is calculated from the equation

(18) $$h_{\min}=r_{n}\left[1-\sin\left(\mathrm{arctg}\;\;\left(\frac{a_{rn}}{a_{tn}}\right)\right)\right]$$

**Figure 6:**
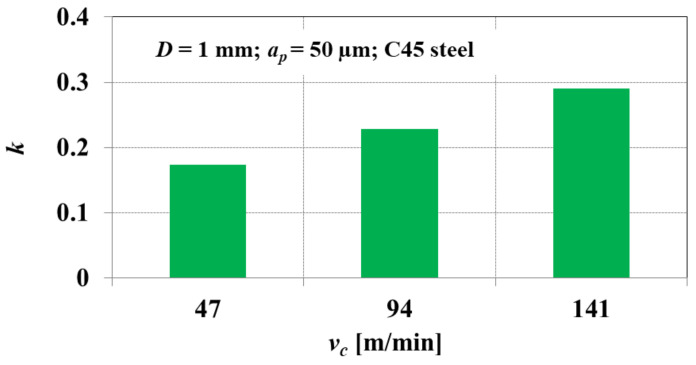
The normalized minimum uncut chip thickness *k* obtained during micro end milling of C45 steel with various cutting speeds *v_c_*. Elaborated on the basis of [35].

Based on the conducted study, the author of [35] demonstrated that during micro end milling of C45 steel, the normalized minimum uncut chip thickness *k* shows an increasing tendency with increasing cutting speed (Figure 6). The obtained dependencies confirm the important role of the heat generated in the cutting zone, in the process of transforming the cut layer into chip. The obtained values of the normalized minimum uncut chip thickness, for the tested cutting speeds, are in the range of 0.17 < *k* < 0.29.

A subsequent group of methods for determination of minimum uncut chip thickness considers the value of the effective rake angle. This angle is dependent on the actual uncut chip thickness *h* and cutting edge radius *r_n_*, which can be described by the following equation:

(19) $$\gamma_{e}=\arcsin\;\;\left(\frac{h}{r_{n}}-1\right)$$

where:

*γ_e_*—effective rake angle.

Wu et al. [36] established the method for minimum uncut chip thickness determination during micro-milling of copper based on the averaging method of the effective rake angle. The authors revealed that minimum uncut chip thickness during micro-milling of copper is equal to 0.17 *r_n_*.

Mikołajczyk et al. [37] proposed a minimum uncut chip thickness calculation method for the oblique turning process of C45 steel, considering the effective rake angle during machining. They formulated the following expression of the minimum uncut chip thickness:

(20) $$h_{\min}=r_{n}\left(1-\frac{1}{\sqrt{\frac{\cos^{2}(k\cdot \lambda_{s})}{\tan^{2}\gamma_{ce}}+1}}\right)$$

where:

*k*—constant defined by the authors, with a values contained within the range between 0.9 and 1;

*λ_s_*—the helix angle;

*γ_ce_*—the effective rake angle in the chip flow direction, determined empirically.

On the basis of the conducted research, the authors have concluded that the value of minimum uncut chip thickness during oblique turning is strongly affected by the tool helix angle. The authors observed that with the growth of the absolute value of the *λ_s_* angle, the minimum uncut chip thickness decreases.

## 4. Simulation/Numerical Methods for Minimum Uncut Chip Thickness Determination

The basis of another group of methods for determining the minimum uncut chip thickness is the simulation of cutting process based on numerical calculations. These approaches mainly use finite element (FEM), molecular dynamics (MD), and smoothed particle hydrodynamics (SPH) methods.

The finite element method is a very popular simulation method applied in machining. Its popularity is primarily related to the possibility of simulating the phenomenon of chip formation, estimating forces, stresses, and temperatures, as well as the size and speed of deformation of the workpiece [38]. In addition to the above-mentioned advantages, the use of FEM enables [39]:

▪ modeling of plane and spatial problems;
▪ the use of various constitutive models of the material and boundary conditions;
▪ obtaining high compliance with experience for both low and high deformation rates;
▪ modeling of continuous (e.g., turning, boring) and interrupted (e.g., milling) cutting;
▪ simultaneous analysis of mechanical and thermal issues.

A literature review shows that the FEM modeling of the minimum uncut chip thickness during machining was based mainly on the use of Johnson–Cook and Bammann–Chies–Johnson (BCJ) constitutive models [40].

Moriwaki et al. [41] used an FEM model based on the Johnson–Cook equation to simulate the chip formation process and *h_min_* during orthogonal micro-cutting of copper with a polycrystalline diamond (PCD) tool. The same model was used by Chen et al. [42] to estimate *h_min_* during the micro-machining of 4340 steel using an end mill with *D* = 0.5 mm.

During precision machining carried out with small uncut chip thicknesses, the traditional Johnson–Cook constitutive model may be characterized by reduced accuracy. The reason for this is the so-called size effect, caused by the occurrence of negative values of the effective rake angles during machining in the range *h* ≈ *r_n_*. The result is an intense increase in compressive stresses in the area of the cutting edge radius, which can lead to a significant strengthening of the workpiece. Accordingly, Lai et al. [43] proposed a modified Johnson–Cook model, taking into account the strain gradient theory of plasticity of the Fleck–Hutchinson model [44] in order to estimate the minimum uncut chip thickness during milling of copper. The developed constitutive model took into account the temperature, size, and rate of deformation of the workpiece, as well as the length of the main slip zone. This model is expressed by the formula

(21) $$\tau_{L}=\tau_{sh}\sqrt{1+{\left(\frac{18\cdot \gamma_{n}^{2}\cdot G^{2}\cdot b_{v}}{\tau_{sh}{}^{2}\cdot l_{\mathrm{sh}}}\right)}^{\rho_{d}}}$$

where:

*τ_L_*—shear stress considering the size effect and length of the main slip zone;

*G*—shear modulus;

*b_v_*—Burgers vector;

*ρ_d_*—constant of proportionality taking into account the total dislocation density;

*l_sh_*—the length of the main slip zone.

The FEM simulations with the use of the developed constitutive model show the presence of plastic flash onto the deformed workpiece during cutting in the range *h* < *h_min_*. The maximum values of stresses during cutting with *h* = 10 µm, generated for the model described by Equation (21), were about 40% higher than the values generated for the traditional Johnson–Cook model. This confirms the significant importance of the size effect during precise cutting.

Guo and Anurag [45,46] pointed out that the main limitation of the FEM methods based on the Johnson–Cook model is the assumption that the processed material is single-phase and homogeneous. Nevertheless, in precision machining with small uncut chip thicknesses, multiphase materials show heterogeneity caused by different plastic properties of the phases and the matrix. Accordingly, Chuzhoy et al. [47,48] applied the Bammann–Chies–Johnson (BCJ) variable plasticity model for FEM modeling of heterogeneous pearlitic cast iron. The research shows that micro-machining of a multi-phase material causes greater fluctuations in the shape of the formed chip than those obtained during the cutting of a single-phase material. The above model was also used by Vogler et al. [49] to estimate *h_min_* during micro-milling of ferrite, perlite, and cast iron.

Simulation of the chip formation process along with the estimation of the minimum uncut chip thickness can also be performed using the molecular dynamics (MD) method. This method uses the Newtonian dynamics rules for atoms, and the Newton equation is solved by numerically integrating the trajectory of each atom [50]. In molecular dynamics, the forces acting on atoms are calculated using potential functions that describe the energetic relationship between atoms, taking into account the bond distance and angle [51]. In this way, MD simulations take into account material characteristics in detail and allow for an accurate estimation of dislocation, specific cutting energy, and crack propagation.

The first research on the use of MD techniques for nano-cutting simulation was carried out by Belak and Stoawers [52] in the late 1980s. The next research by Ikawa and Shimada [53,54] involved MD modeling of orthogonal nanometric cutting of copper with a diamond cutting tool. As part of the experiment, the authors investigated the relationship between the cutting edge radius and the minimum uncut chip thickness, as well as the chip formation process and deformations of the subsurface layer. In the following years, many studies of the nanometric cutting process were carried out using MD methods [55,56]. These works concerned only nanoscale cutting (with thicknesses of cut in the range of several nanometers). This limitation results mainly from the demand for a very large amount of computing power in order to determine the dynamic characteristics of the atoms included in the model. Consequently, traditional MD modeling has limited application in precision machining, carried out in the range of submicrometric and micrometric uncut chip thicknesses.

In order to broaden the scope of application of molecular dynamics methods in relation to cutting with higher uncut chip thicknesses, the MD models were modified. Inamura et al. [57,58] developed a method called variable resolution molecular dynamics (VRMD) to simulate both nano- and microscale cutting processes. This method is based on the so-called renormalization, i.e., defining sets of atoms, which were then treated as single atoms (so-called virtual atoms) by rescaling their size. As part of the research, the authors simulated chip formation during orthogonal cutting of monocrystalline silicon with a diamond cutting tool with *h* = 1 µm. Another approach to simulate MD-based submicrometric cutting is the implementation of a graphics processing unit (GPU) in a computer to assist with numerical calculations. Popular software used for MD modeling (e.g., LAMMPS, Amber, ACEMD, NAMD) enables the use of GPU support during computations. According to Brown et al. [59], this contributes to an over 10-fold increase in computing efficiency in relation to the calculations performed only using a computer processor (CPU). This approach was adapted in the research of Xiao et al. [60]. Their experiment consisted in modeling chip formation during cutting of a brittle SiC with a diamond tool, and the uncut chip thickness *h* = 0.04 µm.

The application of MD has some limitations towards the modeling of chip formation during precision and ultra-precision cutting, which are attributed to very large demand for computational power. On the other hand, the most popular FE methods employed in modeling of machining, based on Lagrangian or Arbitrary Lagrangian Eulerian (ALE) approaches [61,62], also imply some difficulties. In most studies, the simple Coulomb friction model is applied for the description of tribologic aspects of machining. Nevertheless, it can lead to some inaccuracies in modeling affecting the modeled cutting forces or minimum uncut chip thickness. The second aspect is the applied workpiece/chip material separation model, which in case of Lagrangian approaches, can induce excessive grid distortions. This limitation implies the application of ALE methods and time consuming remeshing techniques.

In order to overcome these limitations, in a recent years, a novel method based on smoothed particle hydrodynamics (SPH) has gained popularity in the modeling of cutting processes. Smoothed particle hydrodynamics is based on the Lagrangian equation and was initially developed in the 1970s to simulate astrophysical phenomena [63,64]. The SPH approach involves the use of a kernel approximation to derive spatial derivatives within randomly distributed interpolation points. This is a meshless method; thus, the computational grids are not required to carry on the simulations. In SPH, the workpiece material is rather represented as a group of Lagrangian particles, which interact with each other based on a smoothing length and a statistical kernel function. In general, the length of the smoothing affects the individual particle search radius to find neighboring particles. The kernel function, on the other hand, is used to determine how strongly or weakly particles interact [65].

When comparing the conventional FEM modeling of cutting process to the one based on the SPH approach, some advantages of the latter can be indicated. First, it enables modeling of high strains characteristic of the cutting process. Therefore, no remeshing (as in FE methods) is needed. The next advantage of SPH methods is very realistic chip/workpiece separation resulting from the relative motion of particles. The last advantage is no need for definition of the friction coefficient, the value of which results directly from the particles’ interactions. It should be noted here that in the case of FE approaches, the appropriate selection of friction model parameters is essential for a reliable modeling of cutting process. However, the SPH methods also have some disadvantages compared with FE methods. In the case of the standard SPH approach, the chip curve is being underestimated. In order to overcome this difficulty, an additional renormalization procedure is required. Additionally, the application of SPH to the modeling of cutting process can be characterized by the appearance of some numerical instabilities. Espinosa et al. [66] observed the numerical instabilities in calculations of chip morphology. This problem has not been seen only in cases of the cutting process model with very high cutting speed.

In relation to the machining process, the SPH method is usually being employed to model the chip morphology [67,68], cutting forces, and specific cutting force coefficients [69,70,71], as well as the stresses in a cutting zone [72,73]. On the other hand, taking into account the minimum uncut chip thickness, the SPH method was applied by Guo et al. [74] to simulate the oxygen-free copper micro-cutting process. The authors conducted the simulation for a different chip thickness to cutting edge radii ratios *h*/*r_n_*. The authors observed that during SPH simulations with the lowest *h*/*r_n_* equal to 0.5, the shearing process was strongly inhibited, and the chip was formed mainly by extrusion of the tool edge. This indicated that the selected *h*/*r_n_* ratio corresponded to minimum uncut chip thickness. Zhao et al. [75] employed the SPH approach to model micro-machining oxygen-free high-conductivity copper (OFHC). It should be noted that their model has been extended by the consideration of the sequential tool passes towards the workpiece. They noticed that value of minimum uncut chip thickness has decreased in a second tool pass, which was attributed to the stress value differences in a machined layer.

## 5. Experimental Methods for Minimum Uncut Chip Thickness Determination

The last group of methods for determining the minimum uncut chip thickness are approaches based on experimental research. Experimental methods enable estimation of *h_min_* both directly—e.g., by assessing the surface topography (understood as a set of surface irregularities in space) and the two-dimensional surface roughness profile of the machined surface—and indirectly by measuring and analyzing physical phenomena correlated with the minimum uncut chip thickness (e.g., measurement of the cutting force components, acoustic emission).

**Figure 7:**
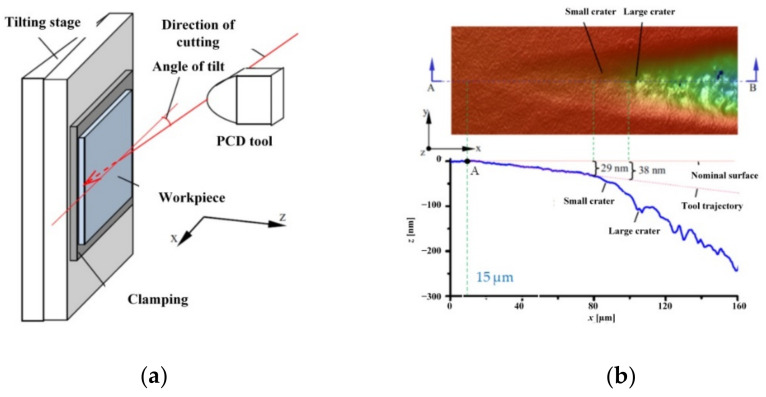
Experimental determination of *h_min_* by measurement of the 3D surface topography of the machined groove: (**a**) experimental setup, (**b**) 3D surface topography and surface roughness profile. Elaborated on the basis of [60].

Xiao et al. [60] assessed the 3D surface topography of brittle SiC obtained by planing a conical groove in order to directly determine the minimum uncut chip thickness and the critical thickness of the brittle–plastic transition *h_pl_*. The tests were carried out on an ultra-precise lathe with a cutting speed of *v_c_* = 1.5 mm/s and with a sample inclination angle at the level of 0.03° (Figure 7a). In order to minimize the influence of the nominal material surface roughness on the obtained results, the sample was polished, and the surface roughness *Ra* ≈ 2 nm was obtained. The analysis of the surface topography of the machined groove (Figure 7b) shows that that in the range of the contact path of the tool with the workpiece, equal to 15 µm, no groove formation was observed. Therefore, point A denoted in the surface roughness profile chart corresponds to the presence of the *h_min_* value. Using the trigonometric dependencies to the measured value of the tool path (15 µm) and the sample inclination angle (0.03°), *h_min_* = 8 nm is obtained. The analysis of the groove bottom surface profile also enables the identification of the critical thickness of the brittle–plastic transition *h_pl_* for the SiC material. In the case of brittle materials, this parameter determines the transition in terms of plastic deformation and brittle cracking. Figure 7b shows that for the uncut chip thickness of 29 nm, there is an intensive increase in the distance of the tool path relative to the profile of the machined surface. This proves the propagation of cracks deep into the material and decohesion as a result of the brittle cracking (thus *h_pl_* = 29 nm), which was also demonstrated by Yan et al. [76].

Liu et al. [22] also used a machined surface topography assessment to determine *h*_min_ during micro-milling of 1040 steel and Al6082-T6 alloy. In their considerations, they assumed that rapid changes in the height of the machined surface roughness result from the transition from the ploughing mechanism to the micro-cutting of the workpiece. Therefore, the maximum heights of these irregularities correspond to the value of *h*_min_. The accuracy of this method may raise some doubts. It needs to be highlighted that the surface irregularities formed during cutting also depend on the kinematic and geometric representation of the cutting edge in the material (feed per tooth and tool diameter), the instantaneous values of the tool deflection, geometric errors of the machining system, and wear of the cutting edge, as well as some random phenomena [77,78,79,80]. Therefore, the estimation of the *h*_min_ value directly on the basis of the surface roughness, without quantifying the contribution of the above-mentioned factors, may be subject to a large error.

In order to determine *h*_min_ on the basis of the surface roughness height, taking into account the influence of the kinematic–geometric model, one can use the Brammertz approach, which has been employed by the authors of [81]. This model makes it possible to determine the theoretical surface roughness *Rzt* for the projection of the cutting edge arc, taking into account the feed per tooth *f_z_*, corner radius *r_ε_*, and *h*_min_ according to the equation [82]

(22) $$Rzt=\frac{f_{z}{}^{2}}{8r_{\varepsilon }}+\frac{h_{\min}}{2}\left(1+\frac{r_{\varepsilon }\cdot h_{\min}}{f_{z}{}^{2}}\right)\;$$

where:

*Rzt*—theoretical surface roughness height;

*f_z_*—feed per tooth;

*r_ε_*—corner radius.

**Figure 8:**
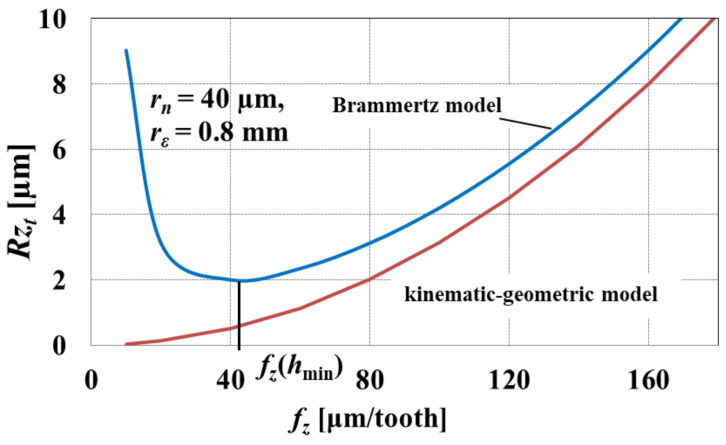
The influence of feed per tooth *f_z_* on the theoretical surface roughness height based on the kinematic–geometric model and model considering the effect of minimum uncut chip thickness. Own elaboration.

The graphic interpretation of Equation (22) is shown in Figure 8. According to Figure 8, there is a local minimum of the function described by Equation (22), the position of which corresponds to the lowest value of the *Rzt* parameter and the value of *f_z_*(*h*_min_). The symbol *f_z_*(*h*_min_) means the feed per tooth corresponding to the minimum uncut chip thickness. Therefore, the determination of the location of the *f_z_*(*h*_min_) value based on the experimentally determined course of the surface roughness height, as a function of the feed per tooth, enables the identification of the *h*_min_. The quantitative relationship between *f_z_*(*h*_min_) and *h*_min_ is described by the following equation:

(23) $$f_{z}\left(h_{\min}\right)=\sqrt{2\cdot h_{\min}\cdot r_{\varepsilon }}\;$$

where:

*f_z_* (*h*_min_)—feed per tooth corresponding to the minimum uncut chip thickness.

After transforming Equation (23), knowing the *f_z_* (*h*_min_) value, one can obtain the expression for the minimum uncut chip thickness:

(24) $$h_{\min}=\frac{f_{z}{\left(h_{\min}\right)}^{2}}{2\cdot r_{\varepsilon }}\;$$

It should be emphasized that the accuracy of this method is strictly dependent on the ratio of the measured value of the minimum surface roughness *Rz*_min_ to the theoretical value of the minimum surface roughness *Rzt*_min_, determined from Equation (22). If *Rz*_min_/*Rzt*_min_ ≈ 1, it can be assumed that the profile of the machined surface is formed as a result of the kinematic–geometric projection of the tool in the workpiece and the phenomenon of the minimum uncut chip thickness. If *Rz*_min_/*Rzt*_min_ ≠ 1, the formation of surface roughness is also determined by other phenomena accompanying the cutting process. Then, the estimation of the minimum uncut chip thickness based on the known value of *f_z_* (*h*_min_) may be characterized by a significant error. It should be noted here that a very important factor affecting the machined surface roughness and minimum uncut chip thickness is progressing tool wear. The changes in tool microgeometry resulting from tool wear can affect the values of an effective tool radius or cutting edge radius. According to Afazov et al. [83], during micro-milling of AISI 4340 steel, the cutting edge radius increased from *r_n_* = 1.5 µm for a new tool to *r_n_* = 25 µm after the micro-milling with a cutting path equal to 600 mm. This observation indicates that during the micro-machining process, even relatively slight tool wear can lead to almost 16-fold growth in the minimum uncut chip thickness.

**Figure 9:**
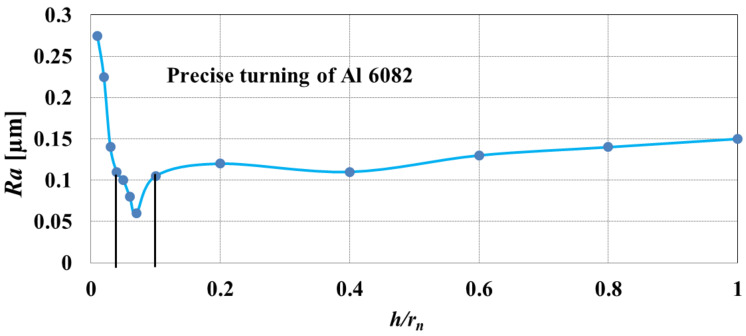
Influence of the *h*/*r_n_* ratio on surface roughness *Ra* during precision turning of Al 6082 alloy. Elaborated on the basis of [84].

The above approach for determining *h*_min_ was used by Rahman et al. [84] during the studies concerning precision orthogonal turning of an Al 6082 alloy with a cubic boron nitride (CBN) tool. The authors examined the surface roughness parameter *Ra* as a function of the ratio of the uncut chip thickness to the cutting edge radius *h*/*r_n_*. The research showed that the lowest value of surface roughness *Ra* was obtained for *h*/*r_n_* = 0.08 (Figure 9). Therefore, the obtained value *h*/*r_n_* = 0.08 represents the normalized minimum uncut chip thickness, denoted by *k*. The above observations were confirmed by chip shape analysis, on the basis of which it was found that for *h*/*r_n_* < 0.08 the obtained chips were incomplete and irregularly shaped, as well as intensely swollen. This indicates the occurrence of the ploughing phenomenon characteristic of cutting in a range of *h* < *h*_min_.

Based on the research carried out by Schoop et al. [85] on the precise milling of porous tungsten, it was established that the method of surface roughness analysis as a function of the uncut chip thickness can also be used to identify the critical thickness of the brittle–plastic transition *h_pl_*.

Oliveira et al. [86] developed a method for determining *h*_min_ based on the measurement of the total force components and machined surface roughness with regard to the micro-milling of 1045 steel. In order to identify the range of occurrence of the minimum uncut chip thickness, they adapted the ratio of the specific cutting force to the machined surface roughness *k_c_*/*Ra*. According to the authors’ assumptions, this coefficient provides information on the intensity of the burnishing phenomenon and the formation of microcracks in the material. Therefore, the area where the sharp increase in the *k_c_*/*Ra* value is observed is related to a reduction in an uncut chip thickness below the *h*_min_ value. It was also demonstrated that for 1045 steel, the range of *h*_min_ corresponds to the values of *k_c_*/*Ra* in the range of 200–280 GPa/µm. This was confirmed by the SEM analysis of the machined surfaces. In the range of *k_c_*/*Ra* ≤ 200 GPa/µm, the surface profile is generated on a regular basis with machining marks, which proves that the shearing process occurs (i.e., the workpiece is being cut). However, in the range of *k_c_*/*Ra* ≥ 280 GPa/µm, surface microcracks and no machining marks were observed, and this is related to the intense burnishing/ploughing of the material, characteristic of *h* < *h*_min_.

Rezaei et al. [87] applied the experimentally determined specific cutting force for the estimation of minimum uncut chip thickness during micro-milling of Ti-6Al-4V alloy in a dry cutting and minimum quantity lubrication (MQL) environment. The authors have stated that a nonlinear increase in specific force coefficient is noticed when the uncut chip thickness decreases, due to the ploughing and size effect, resulting from the third deformation zone, which significantly contributes to the cutting mechanism. Based on the carried out experiment, it was found that a cooling environment directly affects the value of minimum uncut chip thickness. In the case of dry micro-milling, the minimum uncut chip thickness was found to occur at 25–49% of the cutting edge radius, and during micro-milling in MQL conditions, the minimum uncut chip thickness is contained within the range of 15–34% of the cutting edge radius. 

Yun et al. [88] carried out measurements of the total force components during copper micro-milling in order to identify the area of the ploughing phenomenon and the minimum uncut chip thickness. They assumed that in the range of machining with *h* < *h*_min_ that there are significant oscillations of the force values associated with intense ploughing of the material. Therefore, in order to estimate the range of *h*_min_ occurrence, they determined the normalized variance of the peak values of the resultant cutting force s^2^ (*F_N_*) according to the equation

(25) $$s^{2}\left(F_{N}\right)=\frac{s^{2}\left(F_{iN}\right)}{F_{iN\mathrm{av}}}$$

where:

*s*^2^ (*F_N_*)—normalized variance of the peak values of the resultant cutting force;

*F_iN_*—*i*-th peak value of the measured resultant force;

*F_iNav_*—mean value of the measured resultant force.

According to the authors of [88], the decrease in the value of the uncut chip thickness *h* below *h*_min_ corresponds to a rapid increase in the value of s^2^ (*F_N_*). The minimum uncut chip thickness during copper micro-milling is obtained for s^2^ (*F_N_*) = 0.0015.

**Figure 10:**
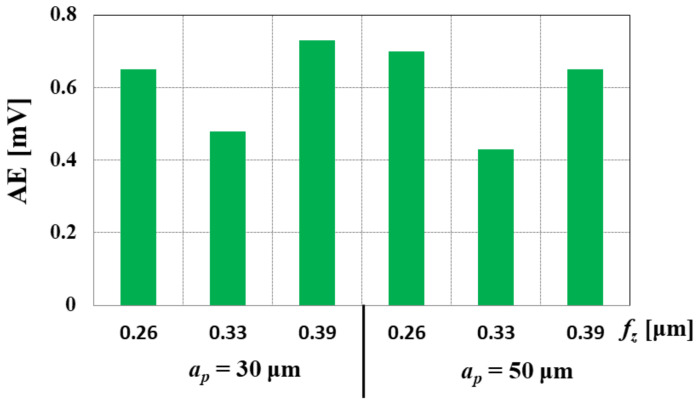
Influence of feed per the tooth *f*_z_ and axial depth of cut *a_p_* on the value of acoustic emission *AE* during micro-milling of electrolytic copper. Elaborated on the basis of [89].

Camara et al. [89] used the measurements of acoustic emission (AE) during micro-milling of electrolytic copper in order to determine *h*_min_. Measurements of acoustic emission enable the assessment of phenomena accompanying the cutting process, such as the formation of the slip plane, crack propagation, reorientation of the grain boundary, or tool wear [90,91,92]. The authors assumed that exceeding the value of the minimum uncut chip thickness (*h* > *h*_min_) would be accompanied by a decrease in the AE value due to the reduction in the intensity of the ploughing phenomenon. The relatively high values of AE signals are probably caused by the periodic instabilities during chip formation in ploughing regime cutting. The conducted research confirmed this hypothesis, and minimum uncut chip thickness was obtained for the feed per tooth of *f_z_* = 0.325 µm/tooth (Figure 10). The increase in AE for higher values of the *f_z_* parameter was related to the increase in the values of shear forces influencing plastic deformations in the slip plane.

Krajewska-Śpiewak and Gawlik [93] applied an acoustic emission signal to estimate the minimum uncut chip thickness during the milling of the titanium (Ti6Al4V) and nickel-based (Inconel 718) alloys. The research that was carried out demonstrated that the use of energy extracted from the AE signal can be successfully applied to the estimation of minimum uncut chip thickness for various workpiece materials. Moreover, the minimum uncut chip thickness during the milling process is also affected by the axial depth of cut and cutting speed.

Gawlik et al. [94] also employed the acoustic emission signal to identify the minimum uncut chip thickness during longitudinal turning of titanium-based alloy WT3 and stainless steel OH17. The authors concluded that the AE signal can be used as an indicator of process decohesion in the real-time of its appearance. It is important to notice that the indicator of a signal energy depends on cutting tool conditions (cutting parameters, tool wear), which in turn affect the value of the *h*_min_.

## 6. Summary of Research towards the Minimum Uncut Chip Thickness

**Figure 11:**
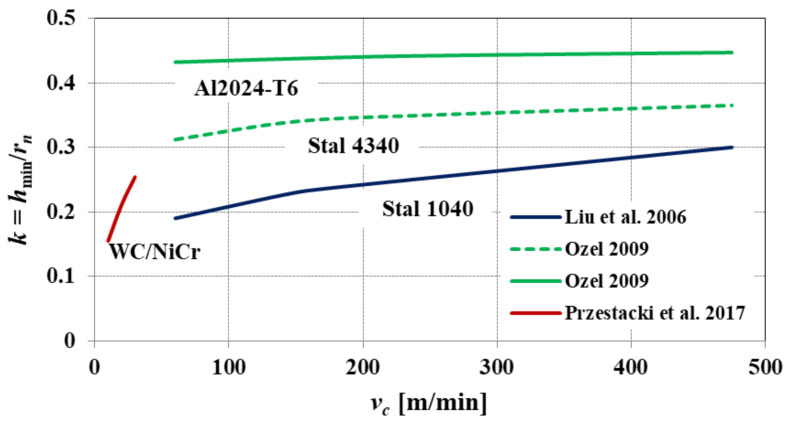
The influence of the cutting speed *v_c_* on the normalized minimum uncut chip thickness *k*, developed based on [22,27,34].

Based on the review of the minimum uncut chip thickness estimating methods, a summary of the obtained values of the normalized minimum uncut chip thickness *k* is presented (Table 1, Figure 11). Knowing the value of the *k* parameter enables determination of the minimum uncut chip thickness *h*_min_ value based on Equation (1).

Table 1 summarizes the *k* values estimated on the basis of analytical, numerical, and experimental methods for various precision cutting processes, tools, and workpiece materials. Regardless of the method, material, and cutting process kinematics, the obtained values of the normalized minimum uncut chip thickness are within the range of 0.08 ≤ *k* ≤ 0.63.

Based on the analysis of the literature, it was found that the value of the normalized minimum uncut chip thickness depends on the value of the cutting speed *v_c_*. According to the research [22,27,34], an increase in the *v_c_* parameter leads to an increase in the value of *k*, regardless of the type of workpiece material and the cutting method. Increasing the cutting speed *v_c_* causes an increase in temperature in the cutting zone, which is caused by the conversion of almost all of the cutting work generated during machining into heat. The increase in temperature leads to an increase in the degree of plasticization of the material in the shear zones, and this results in an increase in the value of the minimum uncut chip thickness.

## 7. Conclusions and Outlook

The problems of physical phenomena during precision and ultra-precision cutting are currently the subject of research in many research centers. These works usually concern aspects of the minimum uncut chip thickness, as well as the dynamics of the precision machining process. Based on the conducted critical review on the minimum uncut chip thickness estimation during precision, ultra-precision, and micro-machining processes, the following conclusions can be drawn:

- Based on the literature review, it is possible to divide the methods of estimating the minimum uncut chip thickness into experimental, analytical, and numerical ones. The use of the approaches from the first group is not an easy task due to the need to carry out precise measurements of the topography of the machined surface and then to recognize the transition area between the ploughing of the material and the shearing (cutting of material). Analytical methods, in turn, require the identification of many material and thermomechanical parameters. They are usually based on complex numerical calculations. An alternative to conventional models can be numerical/simulation approaches due to the relative ease of modeling the cutting process. These models allow not only the simulation of the chip formation phenomenon but also the estimation of force, stresses, and temperatures in the cutting zone.
- Regardless of the approach, the methods used by researchers to estimate the minimum uncut chip thickness mainly apply to orthogonal free cutting (mainly turning). However, there are few models for cutting with tools with a rounded cutting edge profile and oblique process kinematics. It should be emphasized that in practice this type of process kinematics and cutting tool geometries are used very often. According to many studies, the shape of the cutting edge significantly affects the distribution of forces acting on the cutting edge, and also, in the case of some tools (e.g., ball end and torus mills), alternating the values of the cutting speed along the active cutting edge are affected. The variability of these quantities may have a significant impact on the process of the material decohesion and thus the value of the minimum uncut chip thickness.
- During precision, ultra-precision, and micro-milling processes, the chip formation occurs not only by shear along the slip plane but also by elastic–plastic deformations of the workpiece and by certain material properties, such as, e.g., micropores and material fractures, inclusions, and grain boundaries. In addition, during these processes, the microstructure of the workpiece and progressing tool wear can significantly affect the decohesion of workpiece and thus the minimum uncut chip thickness. Therefore, reliable methods for determination of minimum uncut chip thickness in precise machining processes should include the detailed characteristics of the workpiece material, as well as the variable micro-geometry of the cutting edge.
- During micro-milling and precise turning of various metallic materials, independent of selected input process parameters, the normalized minimum uncut chip thickness is contained within the range of 0.08 ≤ *k* ≤ 0.63.

The subsequent research directions on estimating minimum uncut chip thickness should take into consideration:

- more complex oblique cutting processes carried out with tools equipped with various cutting edge contours (e.g., milling with ball end, or torus end mills);
- additively manufactured construction materials, as well as materials with multi-phase microstructure;
- progressing tool wear affecting the real profile of the cutting edge and thus variations in cutting edge radius.

## Figures and Tables

**Table 1:** Summary of research towards determination of normalized minimum uncut chip thickness *k*.

Authors	Year	Method	Workpiece	Technology	*k* = *h*_min_/*r_n_*
Jankowiak et al. [95]	1993	analytic	steel 45, steel 1.3505	turning	0.15–0.63
Yuan et al. [96]	1996	experimental	Cu–Mg–Mn alloy	turning	0.2–0.4
Kim et al. [97]	2004	experimental	brass 360	micro-milling	0.3
Vogler et al. [49]	2004	FEM	ferritic–pearlitic steel	micro-milling	0.14–0.43
Son et al. [31,32]	2005	analytic	aluminum, copper, brass	turning	0.2–0.4
Liu et al. [22]	2006	analytic	steel 1040, alloy 6082T6	turning	0.2–0.4
Lai et al. [43]	2008	analytic	copper	micro-milling	0.25
Woon et al. [98]	2008	FEM	steel 4340	turning	0.26
Ozel et al. [27]	2009	FEM	steel 4340, alloy Al 2024-T6	micro-milling	0.29–0.45
Ding et al. [20]	2011	FEM	steel 45	micro-milling	0.2
Kang et al. [99]	2011	experimental	steel 1045	micro-milling	0.3
Cuba Ramos et al. [100]	2012	experimental	steel 1045	micro-milling	0.29
Malekian, Park, and Jun [33]	2012	analytic	alloy 6061	micro-milling	0.23
Storch and Zawada-Tomkiewicz [30]	2012	analytic	steel C55	turning	0.08
Oliveira et al. [86]	2015	experimental	steel 1045	milling	0.22–0.36
Xiao et al. [60]	2015	MD, experimental	SiC	grooving	0.27
Camara et al. [89]	2016	experimental	electrolytic copper	micro-milling	0.33
Chen et al. [42]	2017	FEM	steel 1045	micro-milling	0.25
Przestacki et al. [34]	2017	analytic	WC/NiCr	turning	0.16–0.25
Rahman et al. [84]	2017	experimental	alloy Al 6082	turning	0.08

## Data Availability

The data presented in this study are available on request from the corresponding author.
